# Full-field, frequency-domain comparison of simulated and measured human brain deformation

**DOI:** 10.21203/rs.3.rs-4765592/v1

**Published:** 2024-08-14

**Authors:** Amir HG. Arani, Ruth J. Okamoto, Jordan D. Escarcega, Antoine Jerusalem, Ahmed A. Alshareef, Philip V. Bayly

## Abstract

We propose a robust framework for quantitatively comparing model-predicted and experimentally measured strain fields in the human brain during harmonic skull motion. Traumatic brain injuries (TBIs) are typically caused by skull impact or acceleration, but how skull motion leads to brain deformation and consequent neural injury remains unclear and comparison of model predictions to experimental data remains limited. Magnetic resonance elastography (MRE) provides high-resolution, full-field measurements of dynamic brain deformation induced by harmonic skull motion. In the proposed framework, full-field strain measurements from human brain MRE in vivo are compared to simulated strain fields from models with similar harmonic loading. To enable comparison, the model geometry and subject anatomy, and subsequently, the predicted and measured strain fields are nonlinearly registered to the same standard brain atlas. Strain field correlations (\(\:{C}_{v}\)), both global (over the brain volume) and local (over smaller sub-volumes), are then computed from the inner product of the complex-valued strain tensors from model and experiment at each voxel. To demonstrate our approach, we compare strain fields from MRE in six human subjects to predictions from two previously developed models. Notably, global \(\:{C}_{v}\) values are higher when comparing strain fields from different subjects (\(\:{C}_{v}\)~0.6–0.7) than when comparing strain fields from either of the two models to strain fields in any subject. The proposed framework provides a quantitative method to assess similarity (and to identify discrepancies) between model predictions and experimental measurements of brain deformation, and thus can aid in the development and evaluation of improved models of brain biomechanics.

## Statements and Declarations

**Author Contributions:** AHGA performed all the simulations, developed methods and concepts, prepared the results, wrote the original manuscript, and reviewed and edited the manuscript. RJO and JDE prepared all the experimental datasets, contributed to the methods, and RJO contributed to the results, reviewed, and edited the manuscript. AJ provided Model 1 and reviewed the final manuscript. AAA provided Model 2, contributed to the results, reviewed and edited the manuscript. PVB supervised and contributed to the methods development and concepts, the results, reviewed and edited the manuscript.

**Funding:** National Institutes of Health (NIH), Grant U01 NS112120.

**Competing Interests:** The authors declare no competing interests.

## 1 Introduction

Traumatic brain injury (TBI) typically results from head impact or acceleration and has a complex pathology due to the cascade of biomechanical factors. These factors typically involve the induced biomechanical forces resulting from head impact and disruptions in neuronal function which may lead to the acute and long-term symptoms including, but not limited to, cognitive deficit (Sahler and Greenwald 2012). One TBI epidemiology study covering New Zealand and North America suggests an annual incidence of between 811 to 979 cases per 100,000 people [2], and another study estimates that TBI affects sixty-nine million people worldwide annually (Dewan et al. 2019). Even mild TBI cases, the most commonly reported category of TBI comprising more than 90% of TBI hospitalizations, can cause acute and long-lasting threats to the cognitive health of patients (Meaney et al. 2014; Okamoto et al. 2019; Maas et al. 2022).

While neuroscience research has focused on biochemical and pathological processes at the cellular level following TBI, strong evidence from recent studies highlights the important role of brain mechanics in the onset and progression of TBI, and its secondary cascades (Goriely et al. 2015; Budday et al. 2020). Important challenges to studying the mechanics of the brain in vivo include: (1) the brain is inaccessible inside the skull; (2) the brain's soft tissue has heterogeneous, nonlinear, anisotropic, poro-viscoelastic properties, with substructures exhibiting dynamic behavior that varies across participants; and (3) actual TBI scenarios cannot ethically be replicated experimentally, because the loading conditions risk harming human participants (Okamoto et al. 2019; Budday et al. 2020; Alshareef et al. 2021a). The first two challenges can be partially addressed by utilizing two noninvasive imaging techniques, magnetic resonance elastography (MRE) and tagged magnetic resonance imaging (tMRI). MRE captures the in vivo response of the brain to external excitation of the skull in a steady-state harmonic motion, and tMRI captures the in vivo response of the brain to mild acceleration of the head induced by an impulsive load (Bayly et al. 2021). A limitation is that non-invasive imaging in live human subjects (Bayly et al. 2021) is only able to measure deformations much smaller than those in injury.

Despite the inherent limitations of in vivo brain experiments, MRE and tMRI have enabled important advances in the experimental quantification of brain mechanics. MRE outputs displacement vector fields in 3D throughout the brain, with typical voxel resolution of 2-3 mm. Dynamic deformation of the brain can be separated from the rigid-body ("bulk") motion by well-established methods (Okamoto et al. 2019). MRE is used primarily as a non-invasive method for estimating soft tissue properties in vivo, especially for organs that are otherwise inaccessible, such as the human brain. Clinically, MRE has existing and potential applications in diagnosis (Okamoto et al. 2019, 2023; Murphy et al. 2019; Arani et al. 2021; Bayly et al. 2021; Sack 2023). In the context of brain mechanics, MRE captures the dynamic deformation of the brain induced by steady-state harmonic motion of the skull, which can be related to the brain's response to an impact on the skull, as imaged in vivo with tMRI (Escarcega et al. 2023).

The challenges of experimental studies make computational brain models attractive for investigating the biomechanical factors leading to TBI. Participant-specific brain models and group-specific templates have been developed and used recently to predict brain mechanics in different loading scenarios, including excitations or impacts at injurious levels that are otherwise impossible or unsafe to acquire in experiments involving human participants (Ji et al. 2014, 2022; Li et al. 2021a). To be useful, computational models require appropriate material models for different regions of the brain, reasonable estimates for parameters, and quantitative assessment of the accuracy and uncertainty of their predictions.

Many brain modeling studies focus on time-domain simulation of head impacts that predict global strain metrics such as maximum principal strain (MPS) as indicators of injury, and compare those predictions to data, e.g., from cadaver head impact tests (see Giudice et al., 2019; X. Li et al., 2021; Trotta et al., 2020). Madhukar & Ostoja-Starzewski (2020) developed a finite element (FE) head model with material properties from nonlinear inversion algorithm of MRE data. They compared the intracranial pressure predicted in simulations to measurements from cadaver impact experiments performed by Nahum et al. (1977) and the displacement values to measurements from Hardy et al. (2001). In another study, by Alshareef et al. (2021a), the authors developed participant-specific brain models with calibrated linear viscoelastic (LVE) material properties determined from MRE experiments. They compared the model predictions of strain percentiles, MPS, and the maximum axonal strain during induced neck rotation and neck extension to the analogous metrics from tMRI experiments on the same participants. They found a closer agreement between simulation-predicted and experimental strain patterns for in-plane strain components than for MPS and out-of-plane components. In a frequency-domain study involving MRE, Y. Li et al. (2021) compared strain fields from simulation and experiments, using different quantitative metrics and LVE model calibration.

In these efforts, the brain modeling community has relied extensively on small set of cadaver head experiments to evaluate the accuracy of the brain models (Ji et al. 2014; Giudice et al. 2019). Model predictions of brain displacement have often been compared to measurements by Hardy et al. (2001) of relative brain/skull motion and brain displacement during cadaveric head impact, using neutral density targets (NDTs) implanted in the brain. Other important studies were performed by Nahum et al. (1977) and Nahum & Smith (1976), who measured intracranial pressure in cadaver head impact experiments.

Discrepancies remain in the predictions of strain fields from different models, even those that have been evaluated using the same set of experiments, and simulated using the same loading conditions (Ji et al. 2014; Giudice et al. 2019). Prior work by Giudice et al. (2019) highlights factors that might explain discrepancies between strain predictions from different models. These authors note the limited evaluations of models by comparison to experimental strain fields. Most cadaveric studies include data from only a sparse set of locations, which precludes accurate strain estimates. Cadaveric brain tissue also differs from live brain tissue, as highlighted by Ji et al. (2014), so FE models evaluated based on cadaveric data may not accurately predict strain in the intact, living human brain. Using kinematic inputs for models based on head impact data from hockey games, Ji et al. (2014) found significantly different predictions from three different computational models. Miller et al. (2017) investigated displacements at specific locations in six evaluated models corresponding to the approximate location of NDTs in experiments by Hardy et al. (2001). They used CORA (CORrelation and Analysis) to rate the performance of the models. While several of the models predicted displacement values similar to those observed in the cadaveric brain, their ability to accurately predict the strain field in the human brain in vivo remains in question.

In this work, we propose quantitative metrics and an underlying framework to compare simulated 3D strain tensor fields to corresponding strain fields measured in the human brain by MRE in vivo. This comparison is performed in the (temporal) frequency domain, over the entire spatial extent of the brain. We employ the concept of cosine similarity of complex vectors to quantify the similarity of data arrays in any domain of interest. This approach relies on image registration to achieve spatial correspondence between predictions from models with different geometries and element types, and data from experiments in individuals with different anatomies. We also test our methods by quantifying the similarity of simulated and measured data in the simpler case of a cylindrical gel “phantom” (physical model).

## 2 Methods

Two previously evaluated FE models of the human brain were used in this study. The model inputs were harmonic head kinematics from MRE experiments in six participants (Section 2.1). The simulation-predicted strain field from each model was compared to the experimentally measured strain field from each participant; metrics based on the tensor inner product were used to quantify similarity and strain magnitude (Section 2.6). We validated the method by comparing strain fields measured in a gel phantom to those predicted by a computational model of the phantom (details on gel phantom comparison are provided in Online Resource Section S1). All simulations were performed in ABAQUS (v. 2021, Dassault Systemes Simulia Corp.), and all quantitative comparisons were performed using custom codes in MATLAB (R2023a, MathWorks Inc., Natick, MA).

### 2.1 MRE experiments

Experimental MRE datasets for human volunteers were acquired from scans performed on a Siemens 3T Prisma MRI scanner at Washington University in St. Louis using an echo-planar imaging sequence (Okamoto et al. 2023). All experimental studies were approved by the Institutional Review Board of Washington University in St. Louis. The data used in this study are available on the Neuroimaging Tools and Resources Collaboratory site (Bayly et al. 2021).

Datasets from the scans of three male and three female healthy young adult volunteers (with no history of brain trauma) were selected for this study. The dynamic brain deformations in these six participants are due to the lateral excitation of the skull by an actuator attached to the right temple of each volunteer; the actuator is connected by plastic tubing to an acoustic driver (Resoundant, Rochester, MN). The lateral actuator generated harmonic skull motion at 20, 30, and 50 Hz. Skull motion primarily consist of right-to-left translation and axial plane rotations; total displacement at any point in the skull is typically <100μm and strains in brain tissue are <0.001 which is well within the low-strain regime (Okamoto et al. 2023).

The rigid-body displacements and rotations estimated from MRE experiments–see Okamoto et al. (2023)–as well as the participants' information, are listed in the Online Resource Section S2, Table S1 and Table S2. The image volume covered by MRE scans was 240×240×132 $mm^3$ with a 3 mm isotropic voxel resolution. The displacement data from the six human participants were Fourier transformed in time and differentiated with respect to space to obtain complex-valued strain fields (Okamoto et al. 2019).

### 2.2 Computational models

The first FE model ("Model 1") was based on a model previously developed and evaluated by comparison to experiments by Garcia-Gonzalez et al. (2017). This model was developed to investigate the effectiveness of two cranial implants, namely polyether-ether-ketone and macroporous hydroxyapatite, in protecting the brain during impact. Since impact response of implants are not the subject of the current study, we assigned skull material properties to implant elements. The second FE model ("Model 2") was previously generated from participant-specific imaging data and evaluated by comparison to deformation fields measured by tMRI in the same participant by Alshareef et al. (2021a), with a participant-specific ID of S01. We have converted Model 2 from ANSYS/LS-DYNA to ABAQUS. Both Models 1 and 2 are shown in **Fig. 1b,c**.

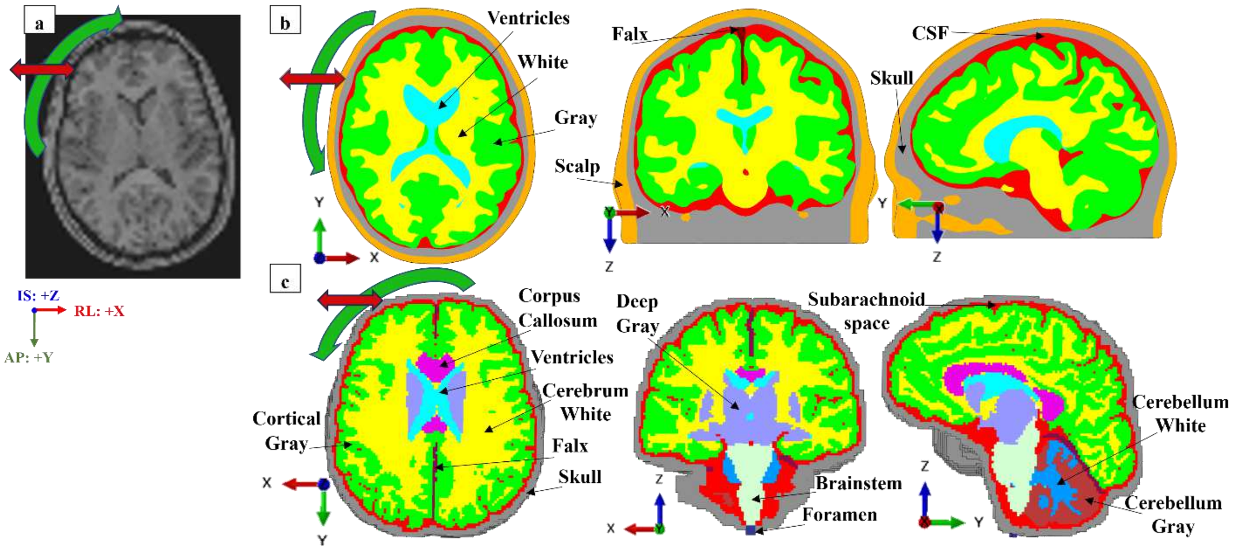

**Fig. 1** Experimental loading directions and model information for two human brain models. (**a)** An axial brain slice in MRE experiment with the participant lying supine with their head inside the head coil. Harmonic motion is induced by a deformable lateral actuator, at 20, 30, and 50 Hz, at the right temple of the participant's skull. (**b**) Model 1 with tetrahedral elements of hybrid type C3D4H in ABAQUS, with seven distinct material regions. (**c**) Model 2 with linear hexahedral elements of hybrid type C3D8H in ABAQUS, with 13 distinct material regions. All coordinate systems are overlaid, and large arrows show the direction of rotation or translation along axes

### 2.3 Material properties

In this work, the material models and corresponding properties of the two brain models were adapted from the original studies to perform benchmark evaluations, using the proposed quantitative comparison framework at the corresponding loading conditions. For the current study, we modified the material properties in Model 1 that were initially used for time-domain simulations (Garcia-Gonzalez et al. 2017). This modification is based on the material properties reported in Table 1 of Y. Li et al. (2021), which are suitable for frequency-domain analysis. This adaptation allows the brain material to exhibit more realistic damping across a wide range of frequencies: the storage and loss modulus both increase with frequency, similar to the trends shown in Fig. 7 from Y. Li et al. (2021). For Model 2, we used previously calibrated material properties for the brain, which exhibit realistic damping over a wide range of frequencies, see Fig. 2 from Alshareef et al. (2021a). Full descriptions of the specific material models and their parameters for Models 1 and 2 are included in the Online Resource Section S3, Tables S3-5. Note that, in both models, the skull is considered as a rigid body; see below.

### 2.4 Simulation of harmonic motion/MRE

A total of 37 simulations were performed using the "Linear Perturbation" procedure in ABAQUS. The frequency-domain analysis provided the complex-valued fields (coefficients of complex exponentials) that described the steady-state response of the models to harmonic excitation at specific frequencies by directly solving the governing equations at physical degrees of freedom. Each brain model was simulated 18 times, comprising six cases for each frequency (20, 30, and 50 Hz) based on the MRE experimental inputs for six participants. For both models, we used a reference point at the centroid of the brain to describe skull excitation. The reference point is tied to the skull elements, which move together as a rigid body following the prescribed rotations and translations defined about the reference point. Since each participant in the experiment has a unique set of kinematic inputs, we ran simulations in ABAQUS using the skull motion of each participant at 20, 30, and 50 Hz. The outputs from these simulations of the two models are complex-valued strain tensors representing the harmonic strain at the centroid of each element.

### 2.5 Nonlinear registration

To compare strain fields between models and experiments of different brain size, shape, and anatomy, it is necessary to transform the strain fields to a common standard space (Escarcega et al. 2023). The standard brain template that we used for registration in this work is based on a part of the International Consortium for Brain Mapping database, which involved 152 young normal adult participants at the Montreal Neurological Institute (Fonov et al. 2011): the "MNI-152" template. Here, we used a version of the T1-weighted MNI-152 nonlinear asymmetric template, upsampled to 0.8 mm resolution, with dimensions of 241×286×241 voxels.

The first step in this process is to construct a pseudo-anatomical (PA) image from the FE models. To create the PA image for Model 1 with tetrahedral elements, the model data points were interpolated onto a 240×240×180 grid of 1 mm isotropic voxels (see Online Resource Fig. S2). The next step was to generate a binary mask, initially in the MNI-152 atlas space, and to perform a reverse mask registration to bring the mask to the Model 1 space. Then the transformed mask is applied to the PA image of Model 1 to remove the non-brain regions generated by interpolation.

Since Model 2 contained only eight-node brick or hexahedral elements, no interpolation was performed, and we reconstructed the mesh in MATLAB using element and node definitions directly imported from ABAQUS (see **Fig. 2a-c** for Model 2). The PA image in this case was reconstructed on a three-dimensional grid with a 1.5 mm voxel resolution (see **Fig. 2b**).

In the next steps for both models, following the approach described by Escarcega et al. (2023), we registered PA images and simulation strain images to the MNI-152 atlas space, where all the MRE strains are transformed. Using the rigid, affine, and SyN transformation algorithms of the antsRegistration code from ANTs software (Avants et al. 2011), the PA images were nonlinearly registered to the MNI-152 atlas.

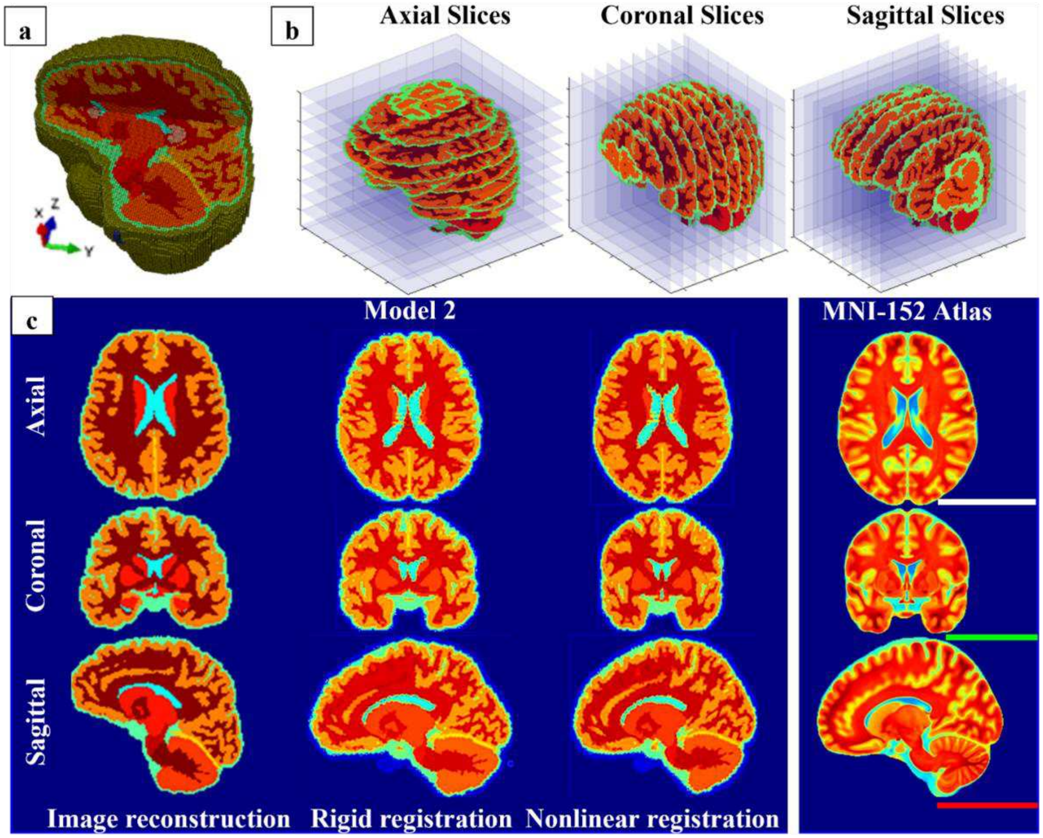

**Fig. 2** Reconstruction and registration of the pseudo-anatomical image of Model 2 to the MNI-152 atlas. (**a**) Model 2 with eight-node brick elements in ABAQUS. (**b**) Centroids of Model 2 elements reconstructed on a three-dimensional grid of 105×117×109 voxels $(x, y, z)$ with 1.5 mm voxel resolution, using a custom MATLAB code that involves no interpolation. (**c**) Model 2 image in three columns: 1) before registration, 2) after rigid registration, now aligned globally with the MNI-152 atlas, 3) after nonlinear registration involving ANTs affine and SyN registration algorithms. The rightmost column shows the target image in this paper, which is the MNI-152 atlas image volume of 241×286×241 voxels $(x, y, z)$ at 0.8 mm voxel resolution. The scale bars in white, green, and red represent 10 cm in axial, coronal, and sagittal views, respectively in MNI-152 space

### 2.6 Comparison metrics

We developed three comparison metrics that measure the magnitudes and similarities of the strain field, with tunable spatial resolution.

#### 2.6.1 Strain magnitude

The real-valued, harmonically varying strain tensor $\boldsymbol{\varepsilon}(\boldsymbol{x}, t)$ at location $\boldsymbol{x} = (x, y, z)$ can be expressed in terms of complex exponential functions with a frequency of $\Omega$ as

$$\boldsymbol{\varepsilon}(\boldsymbol{x}, t) = \boldsymbol{\varepsilon}_0(\boldsymbol{x})e^{i\Omega t} + \boldsymbol{\varepsilon}_0^*(\boldsymbol{x})e^{-i\Omega t} \quad (1)$$

where the complex tensor in Cartesian coordinates, $\boldsymbol{\varepsilon_0} = \begin{bmatrix} \varepsilon_{xx0} & \varepsilon_{xy0} & \varepsilon_{xz0} \\ \varepsilon_{xy0} & \varepsilon_{yy0} & \varepsilon_{yz0} \\ \varepsilon_{xz0} & \varepsilon_{yz0} & \varepsilon_{zz0} \end{bmatrix}$, contains the coefficients of $e^{i\Omega t}$ at the location $\boldsymbol{x}$. In a discretized image volume of dimension $P \times Q \times R$, the complex strain tensor at location $\boldsymbol{x}(I, J, K)$ is stored in array $\boldsymbol{E}_0$, so that $\boldsymbol{E}_0(I, J, K) = \boldsymbol{\varepsilon_0}(\boldsymbol{x}(I, J, K))$ is the second-order strain tensor at that location, in terms of its nine Cartesian components.

We calculate the strain magnitude ($SM$) for every voxel as the root-mean-square value of the norm of the strain tensor $\boldsymbol{\varepsilon}(\boldsymbol{x}, \boldsymbol{t})$ in that voxel. The $SM$ metric quantifies the amount of deformation of tissue at location $\boldsymbol{x}(I, J, K)$ in the image volume. Strain magnitude is estimated from the square root of the tensor inner product of the complex-valued strain tensor with its complex conjugate, i.e., the strain magnitude in a given voxel is:

$$\boldsymbol{SM}(I, J, K) = \sqrt{\boldsymbol{\varepsilon}_0^* : \boldsymbol{\varepsilon}_0} \tag{2}$$

where $\boldsymbol{\varepsilon_0} = \boldsymbol{\varepsilon_0}(\boldsymbol{x}(I, J, K)) = \boldsymbol{E}_0(I, J, K)$**.** The resulting values of $SM$ are stored in a 3D array of size $P \times Q \times R$. Time-averaged octahedral shear strain ($OSS$) is another metric of strain magnitude defined by McGarry et al. (2011); $OSS$ is similar, but not identical, to $SM$ (further reviewed in the Discussion section).

#### 2.6.2 Strain field correlation value

The strain field correlation value ($C_v$), is a metric of the degree of similarity between two 3D strain fields. We introduced this metric to compare experimental strain fields from MRE and tMRI in Escarcega et al. (2023). $C_v$ is a scalar value between 0 and 1, where $C_v = 1$ represents the highest similarity possible (two identical fields) and $C_v = 0$ describes uncorrelated fields. $C_v$ is computed from the sum over all the shared voxels of the tensor inner products of two normalized strain fields, identified by superscripts $q = 1,2$. Before computing $C_v$ , first we create a binary shared strain mask $M_0(I,J,K)$ where voxels in both datasets have computed strain values, through elementwise multiplication of the simulation strain mask $M_1(I,J,K)$ and a participant-specific strain mask $M_2(I,J,K)$; thus $M_0 = M_1 \circ M_2$. Each strain data vector is then normalized throughout the entire shared domain. Following the notation used to define *SM*, we define each of the normalized strain fields as:

$$\widehat{\boldsymbol{E}}_0^{(q)}(I,J,K,S) = \frac{M_0(I,J,K) \circ \boldsymbol{E}_0^{(q)}(I,J,K,S)}{\sqrt{\sum_{I=1}^{P}\sum_{J=1}^{Q}\sum_{K=1}^{R}\left(M_0(I,J,K) \circ SM^{(q)}(I,J,K)\right)^2}}, \qquad q = 1,2 \tag{3}$$

To simplify calculation of $C_v$, we rearrange each normalized strain tensor array $\widehat{\boldsymbol{E}}_0^{(q)}(I,J,K)$ into a normalized strain data "vector" $\overline{\boldsymbol{E}}_0^{(q)}$ of size $(9N \times 1)$, where $N$ is the total number of non-zero voxels in the shared strain mask $M_0$. For a specific voxel at location $\boldsymbol{x}(L) = \boldsymbol{x}(I(L), J(L), K(L))$, the normalized local strain "vector", $\overline{\boldsymbol{\varepsilon}}_0^{(q)}$, is a $9 \times 1$ column array

$$\overline{\boldsymbol{\varepsilon}}_0^{(q)} = \left[\hat{\varepsilon}_{xx0}^{(q)}\ \hat{\varepsilon}_{xy0}^{(q)}\ \varepsilon_{xz0}^{(q)}\ \hat{\varepsilon}_{xy0}^{(q)}\ \hat{\varepsilon}_{yy0}^{(q)}\ \hat{\varepsilon}_{yz0}^{(q)}\ \hat{\varepsilon}_{xz0}^{(q)}\ \hat{\varepsilon}_{yz0}^{(q)}\ \hat{\varepsilon}_{zz0}^{(q)}\right]^T. \tag{4}$$

Thus, the global strain data vector is constructed as

$$\overline{\boldsymbol{E}}_0^{(q)} = \left[\overline{\boldsymbol{\varepsilon}}_0^{(q)}\big(\boldsymbol{x}(1)\big)\ \ \overline{\boldsymbol{\varepsilon}}_0^{(q)}\big(\boldsymbol{x}(2)\big)\ \dots\ \overline{\boldsymbol{\varepsilon}}_{\boldsymbol{0}}^{(q)}(\boldsymbol{x}(N))\right]^T, \quad q = 1,2 \tag{5}$$

and subsequently $C_v$ can be expressed as the complex dot product of the two global strain vectors:

$$C_v(\boldsymbol{E}_0^{(1)}, \boldsymbol{E}_0^{(2)}) = \left\|\overline{\boldsymbol{E}}_0^{(1)*} \cdot \overline{\boldsymbol{E}}_0^{(2)}\right\|;\ C_v : (\mathbb{C}^{P\times Q\times R\times 9}, \mathbb{C}^{P\times Q\times R\times 9}) \to [0, 1]. \tag{6}$$

Here, the superscripts (1 or 2) denote two different strains fields from simulation or experiment. In the above equations, the notations $\boldsymbol{v}^T$, $\boldsymbol{v}^*$, and $\|\boldsymbol{v}\|$ respectively denote the transpose, the complex conjugate, and the magnitude of a complex input $\boldsymbol{v}$ while $\overline{\boldsymbol{v}}$ denotes the normalized vector. The normalization in Equation (3) ensures that $C_v$ remains between 0 and 1, and the more similar the two strain data vectors are, the closer $C_v$ is to 1. The normalization removes global amplitude differences in two strain fields. Therefore, as indicated by Equation (6), $C_v$ is a map from two complex strain fields of size $P \times Q \times R \times 9$ to a scalar between 0 and 1.

#### 2.6.3 Strain field local correlation

While $C_v$ shows how similar two strain fields are in the entire domain of comparison, the same concept can be used at smaller scales to reveal similarities of strain fields for sub-regions within the brain. To capture field correlations between different brain regions, we define the strain field local correlation ($LC$), computed at voxels within the shared strain mask. $LC$ provides an image of similarities with tunable resolution for a domain defined by the shared strain mask in the MNI-152 space. The $LC$ calculation applies a template or cubic mask of $T$ voxels centered on a particular voxel and computes the similarity of the strain fields within the cubic region using the equations defined in Section 2.6.2 for $C_v$, but with the size of the strain data vectors being $9T^3 \times 1$ and assigns $LC$ to the center voxel of the cubic mask in a third array of the same size and dimension as MNI-152 image volume. This process is repeated for all voxels in the image volume.

The process is illustrated in **Fig. 3**; we name the two cubes that move simultaneously through the two datasets and pass through the box filters "Data kernels" or "D-kernels." We denote the arrays that are used in local correlation computations as "Computation kernels" (or "C-kernels"), while the fixed array that stores the results is called the "$LC$ image". $LC$ values stored in C-kernels can be described by the following equation:

$$D^{(q)} = (2l+1)^3 \; B[m,n,p] \cdot \boldsymbol{E}_0^{(q)}[I+m, J+n, K+p], \qquad q = 1,2;$$
$$\{m,n,p\} = -l, -l+1, \dots, 0, \dots, l-1, l, \tag{7.a}$$

$$LC(I,J,K) = C_v(D^{(1)}, D^{(2)}). \tag{7.b}$$

Here, $B$ and $D$ are the box filter and strain data kernel (D-kernel) with a length of $l$ voxels spanning in each direction, respectively. $\boldsymbol{E}_0^{(q)}$, as expressed in Equation (5), is the complex-valued array of strain tensors where the superscript $(q)$ can indicate either a simulated or experimental strain field. The box filter $B$ has a volume of $V = T^3 = (2l + 1)^3$ voxels with a value of $\frac{1}{(2l+1)^3}$ at each voxel. For this study, we chose a value of $l = 8$ voxels (D- and C-kernels 17×17×17 voxels) with 0.8 mm voxels. This results in a sufficiently large filter size (13.6 mm in each direction) compared to the resolution of the models and MRE experiment to span a few elements or voxels of strain data in each spatial direction. The size of the filter is also less than the wavelength of shear wave propagation at the frequencies used in this study. For instance, for a (conservatively low) estimated shear wave speed of 1 m/s, at the highest frequency of 50 Hz the kernel size is smaller than the wavelength of 20 mm.

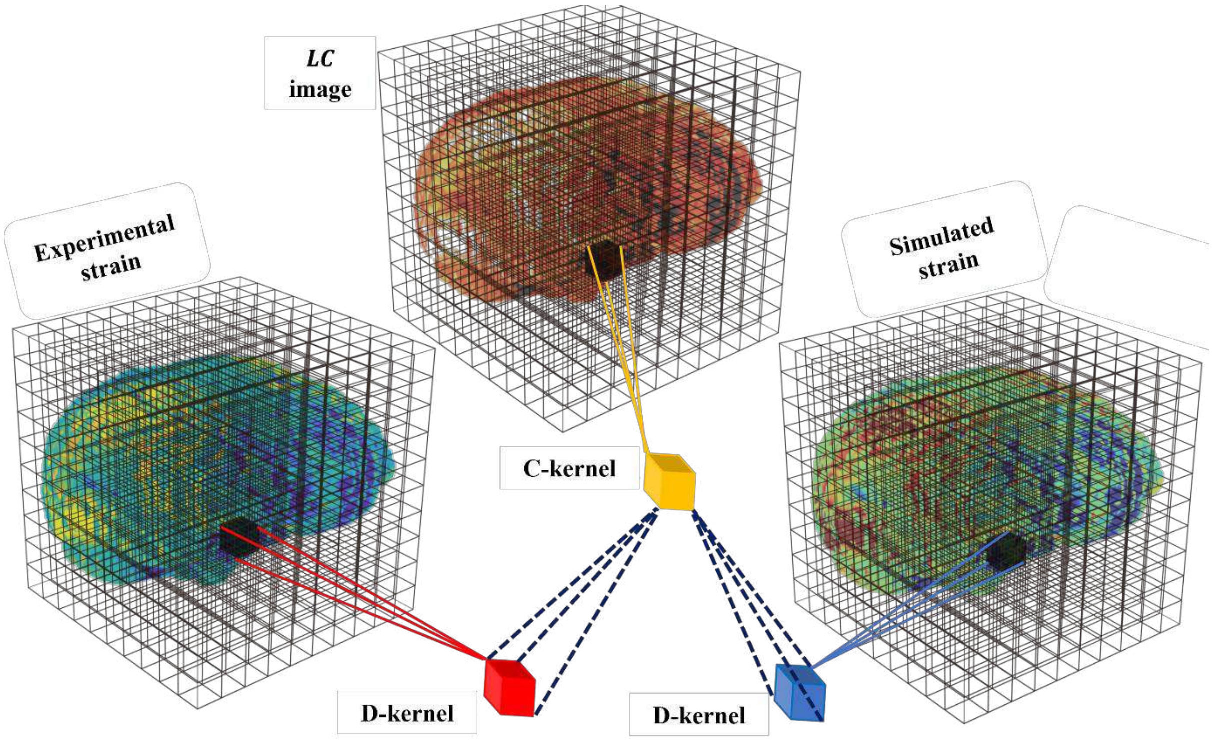

**Fig. 3** Demonstration of local correlation ($LC$) computation. D-kernels are sub-volumes of local strain fields from two different datasets. The size of the objects and arrays, strain patterns, and correlation shown are only for demonstration; they do not represent the actual size or results

## 3 Results

We performed a total of 36 simulations using the frequency-specific experimental excitation inputs from six participants (P01 to P06) as inputs to simulations of harmonic skull and brain motion. Information about participants and the imposed rigid-body motion of their heads are in Online Resource Section S2.

### 3.1 Strain patterns in MRE experiments and simulations

**Fig. 4** shows strain patterns for a representative MRE experiment using human volunteer P01 and the two head model simulations for harmonic motion with frequencies of 20, 30, and 50 Hz. Since the complex strain data in the MRE experiment and model cases had different phase angles, we shifted the phase of the model cases to match the experimental phase. The phase-matched in-plane shear strain $Re(\varepsilon_{xy})$ was normalized in both the brain simulations and the experiment. The real part of the complex field represents a "snapshot" of the harmonically-varying field at zero phase. The qualitative displays of strain patterns show that the wavelength decreases at higher frequencies.

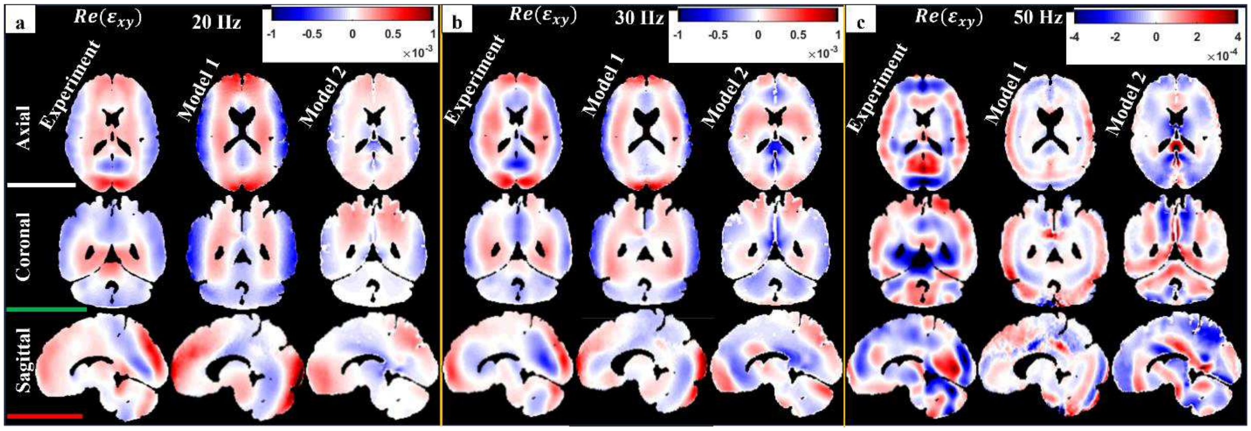

**Fig. 4** Strain patterns for harmonic motion in the brain of human participant, from MRE experiment in subject P01 (Experiment) and simulations of two head models (Model 1 and Model 2). (**a-c**) Normalized and phase-matched shear strain component, $Re(\varepsilon_{xy})$, based on experimental phase angles at 20, 30, and 50 Hz, respectively. The white, green, and red scale bars represent 10 cm respectively in axial, coronal, and sagittal views

In the Online Resource Figs. S3 and S4, we provide qualitative results for all components of the strain tensor for the two brain models and participant P01 data.

### 3.2 Comparison metrics

#### 3.2.1 Strain magnitude ($SM$)

Values of $SM$ for registered 3D strain fields from the MRE experiment (participant P01) and two model simulations are shown in **Fig. 5** for 20, 30, and 50 Hz. $SM$ highlights at least two key features of the strain patterns: (1) $SM$ values are typically highest in the experimental data and lowest for Model 1, and (2) values of $SM$ decrease with increasing frequency.

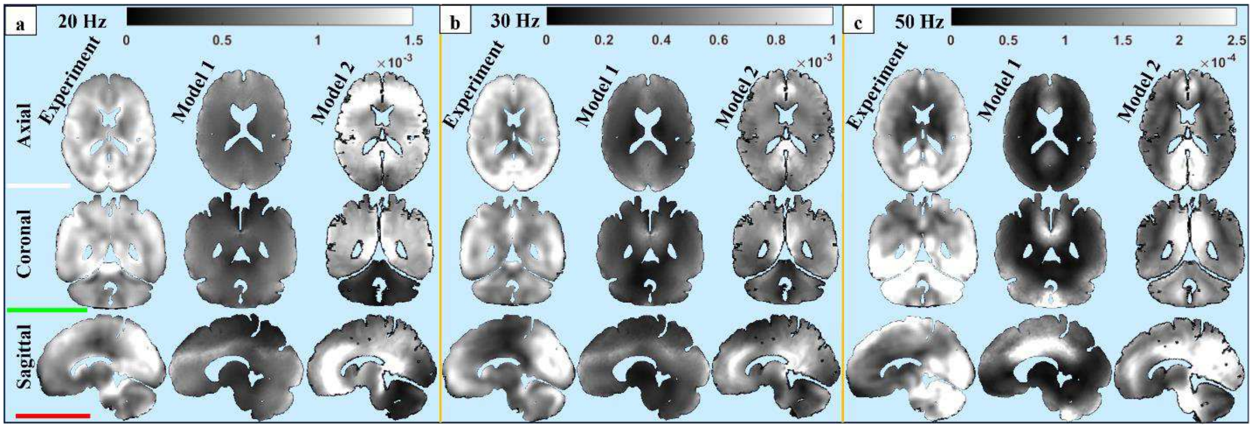

**Fig. 5** Maps of strain magnitude, $SM$, at 20, 30, and 50 Hz for harmonic motion of the skull and brain in a human participant (P01) from MRE experiment and Model 1 and Model 2 simulations. White, green, and red scale bars represent 10 cm in axial, coronal, and sagittal views

#### 3.2.2 Strain field correlations: global ($C_v$) and local ($LC$)

Values of the global strain field correlation ($C_v$) and local correlation ($LC$) were computed between nonlinearly registered 3D strain fields from MRE experiment and each simulation case. Simulation cases were moderately similar ($C_v$~0.4) to the P01 MRE experiment at 20 and 30 Hz (**Fig. 6**) and similarity decreased at 50 Hz. This observation when combined with wave patterns in **Fig. 4** suggests that strain patterns with longer wavelengths tend to have higher similarity. In this specific case, the correlation to experiment is slightly higher in Model 2

than Model 1. The local correlation ($LC$) shows the regions of the brain where simulation predictions deviate from experimental strain measurements.

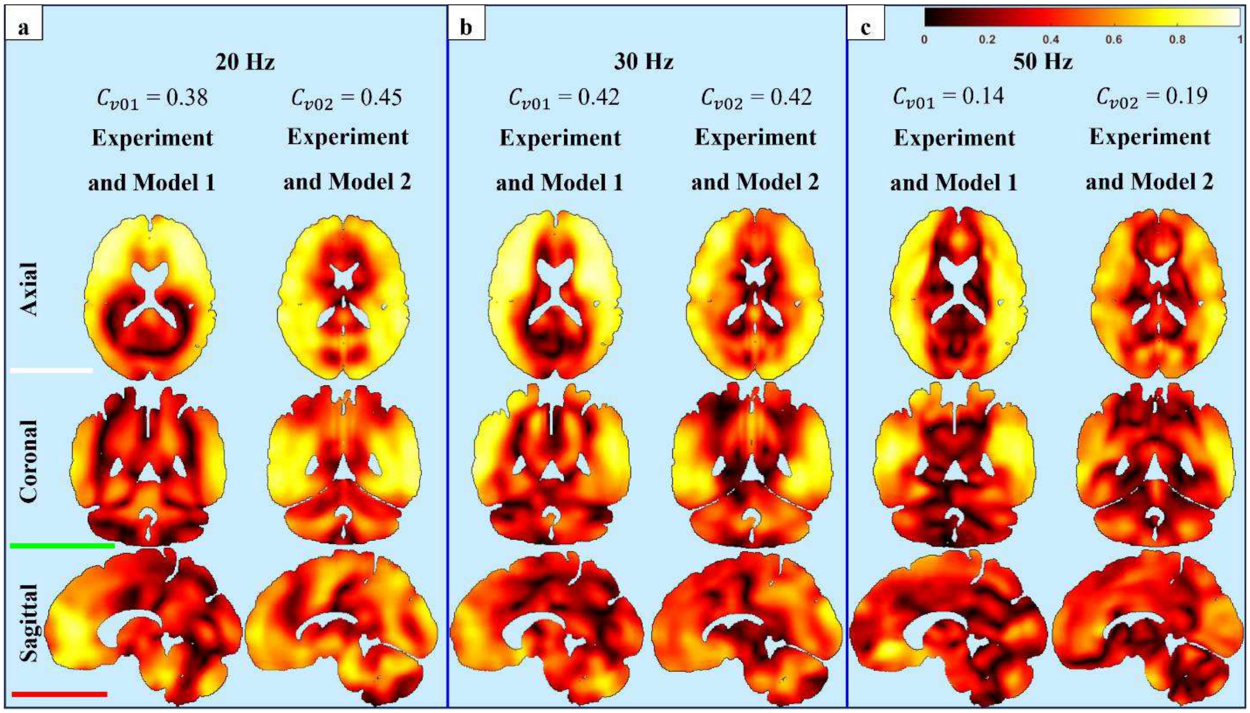

**Fig. 6** Local correlation ($LC$) and global strain field correlation ($C_v$ ) values comparison of strain fields from two models and the P01 MRE experiment. **(a-c)** Calculated $C_v$, and $LC$ for 20, 30, and 50 Hz harmonic motion. White, green, and red scale bars represent 10 cm respectively in axial, coronal, and sagittal views

#### 3.2.3 Cumulative distributions of strain magnitude

To compare the cumulative distributions of strain magnitudes for all six MRE experiments and simulations, the means and standard deviations of the cumulative distributions of $SM$ are shown in **Fig. 7** for 20, 30, and 50 Hz. The cumulative distribution function defined here is the integral of the standard normal distribution of strains that is always increasing in values from 0 to 1; see Gabbiani and Cox (2010) for more details. The $SM$ cumulative distributions in all three cases of the experiment and simulations follow similar trends. However, across all three frequencies, $SM$ is usually the highest in the experiment and the lowest in the Model 1 simulation.

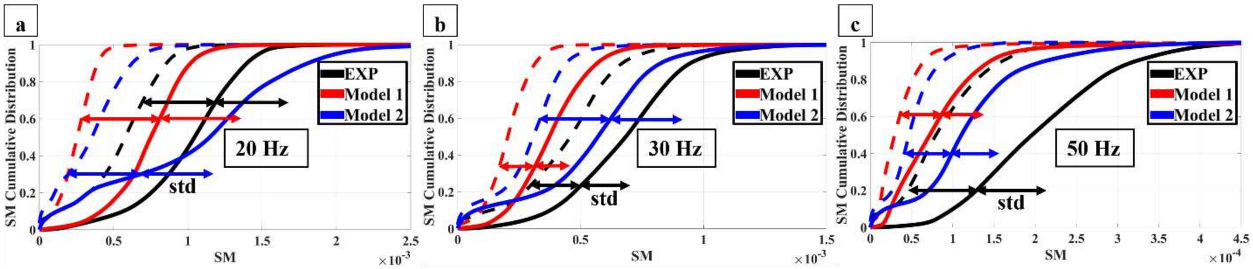

**Fig. 7** Curves of the cumulative distribution of strain magnitude ($SM$) based on fitting to the normal distribution. (**a**) Strain fields from 20 Hz excitation; (**b**) 30 Hz; (**c**) 50 Hz

#### 3.2.4 Local strain field correlations ($LC$) statistics

Statistics (means and standard deviations) of the local correlations between the strain fields from Models 1 and 2 and the six MRE experiments are shown in **Fig. 8**. The first four columns in each panel of **Fig. 8** show the $LC$ statistics for comparisons between all of the six experiments and six simulations of each model with matching excitations. The two right-most columns in each panel show the voxelwise statistics (mean and std. deviation) for the local correlation between each participant's strain field and that of all other participants (15 unique comparisons). In all cases, higher similarities are observed between pairs of experimental strain fields than between strain fields from either model and corresponding experiment.

#### 3.2.5 Global strain field correlation ($C_v$) statistics

Global correlations between strain fields in the brains of six human participants with each other and with strain fields from two different models are summarized by boxplots in **Fig. 9**. For all frequencies, the values of $C_v$ are higher for comparisons between experimental strain fields than for comparisons between simulated and experimental strain fields, or comparisons between the two simulated strain fields. The values of $C_v$ also decrease markedly at 50 Hz for comparisons involving simulations.

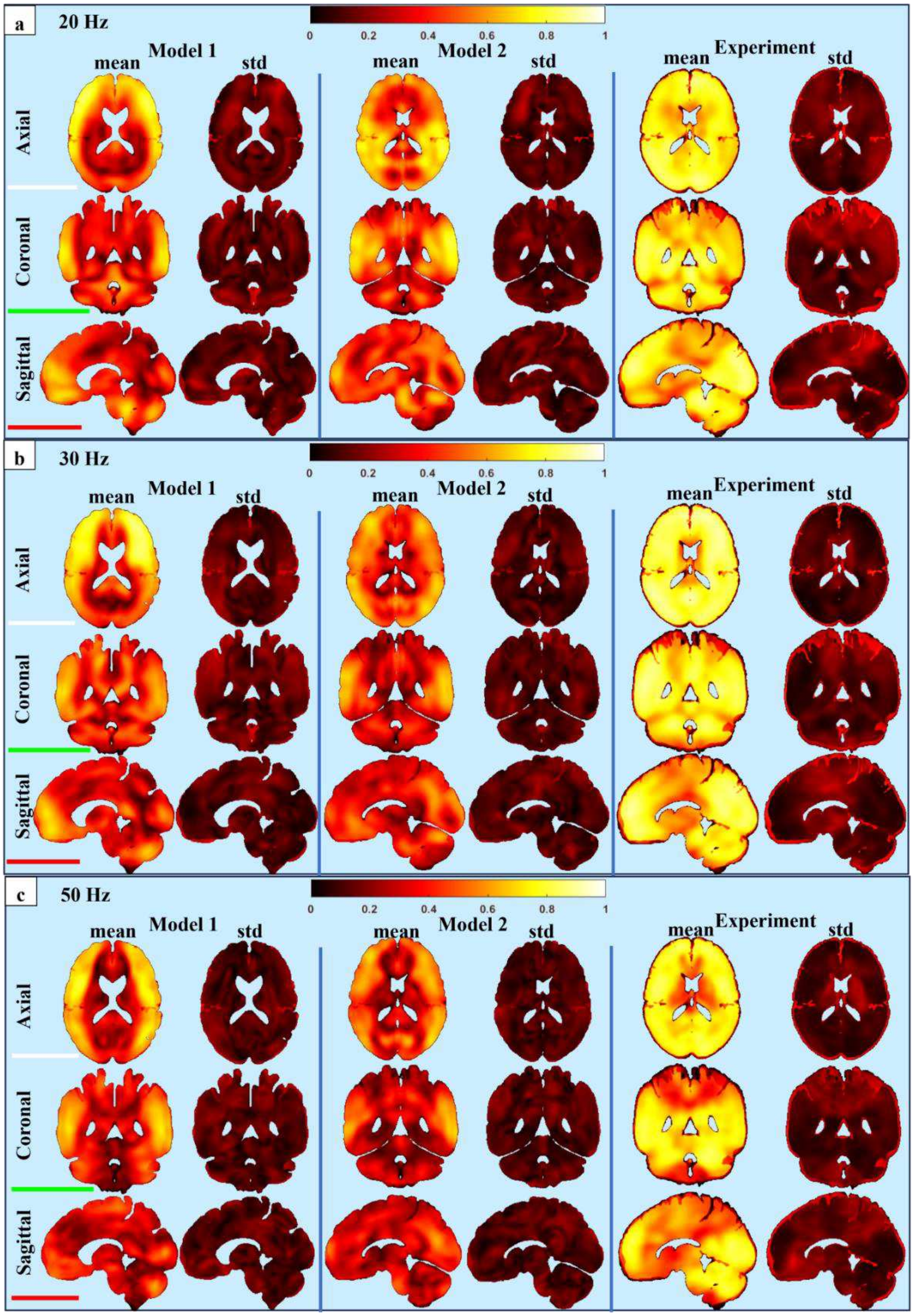

**Fig. 8** Local correlation ($LC$) values for comparisons between strain fields from two models and brain MRE experiments in six participants. Voxelwise mean and standard deviations for six comparisons, between each model simulation and corresponding experiment, are shown for (**a**) 20 Hz, (**b**) 30 Hz, and (**c**) 50 Hz. Statistics from 15 unique comparisons between experimental strain fields are shown in the two right-most columns. White, green, and red scale bars represent 10 cm respectively in axial, coronal, and sagittal views

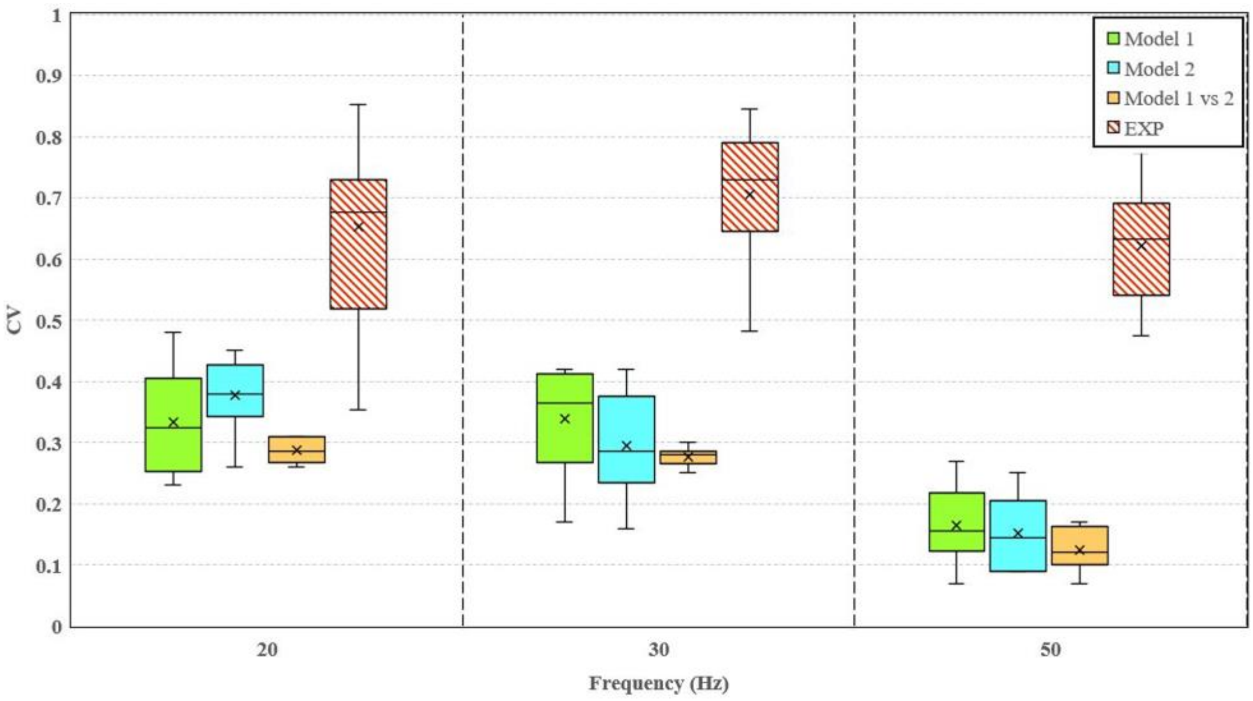

**Fig. 9** Summary of global strain field correlation ($C_v$) for comparisons between strain fields from brain MRE experiments in six participants and strain fields predicted by two models at 20, 30, and 50 Hz. All comparisons were performed between fully registered 3D strain fields. For each box: cross = mean; horizontal line = median, box limits = upper and lower quartiles; end bars = maximum and minimum values. Each box involving Model 1 or 2 or both models contains six unique comparisons; statistics for comparisons between experimental strain fields are based on 15 unique comparisons

## 4 Discussion

The quantitative framework proposed in this study provides a global scalar metric of similarity ($C_v$) and an array of local similarity estimates ($LC$) to objectively compare the similarity between strain fields in the brain excited by harmonic skull motion. The strain magnitude ($SM$) is computed from the norm of the strain tensor at each voxel to quantify the amplitude of deformation. These metrics define a multiscale, frequency-domain comparison framework for quantifying discrepancies between two strain fields. In our comparative study, we compared 3D strain fields from MRE studies in six human participants to predicted strain fields from two computational models.

We found that when we simulated the two models using the same loading conditions as the experimental cases, model predictions shared qualitative features with experimental strain fields but exhibited quantitative differences (**Fig. 8** and **Fig. 9**). This disparity may be due to differences between model geometry and subject anatomy, as well as inaccurate assumptions or approximations. For example, the brain-skull interface, which plays an important role in wave transmission and attenuation of motion, as demonstrated by Badachhape et al. (2017), is not well characterized.

The $LC$ statistics in **Fig. 8** illuminate regional similarities and discrepancies between model predictions and experimental measurements. The maps of $LC$ can reveal underlying issues such as a substructure that has unmodeled effects, or local mesh distortions, inaccurate material properties, or suboptimal segmentation. The current kernel size (17×17×17 array of 0.8mm isotropic voxels) allows several kernels per wavelength of shear wave propagation, and can resolve the effects of anatomical features in the brain. We show $LC$ results with different kernel sizes in Online Resource Fig. S5.

It is also possible to compare predictions of the two models; in this case they do not agree highly with each other when they are subjected to the same harmonic loading (**Fig. 9**). Other studies have noted analogous discrepancies between model predictions for the same kinematic conditions. These brain models were developed for specific purposes not related to the current experiments, and differ accordingly in their geometry, discretization, and calibration (Ji et al. 2014, 2022; Giudice et al. 2019). In this study, material properties were defined in ABAQUS to be evaluated in the frequency domain and the viscoelastic damping of Model 1 was adjusted as described in Section 2.3.

There are a number of limitations to this study. Most importantly, the experiments and simulations involve small-amplitude, harmonic motion typical of MRE experiments (<0.001 strain). We note however, that the strain patterns in MRE are well correlated to strain fields from impulsive loading with deformations larger by approximately two orders of magnitude (>0.05 strain). Although we used two recently developed and evaluated brain models (Garcia-Gonzalez et al. 2017; Alshareef et al. 2021a) in this study, as noted above, material properties of the original models were adapted to corresponding frequency-domain versions, and the damping properties of Model 1 were adjusted.

Brain material properties are a commonly cited source of uncertainty in models, both in this study and in the modeling literature generally (Salahshoor & Ortiz, 2024; Svensson et al. 2021). The lack of a universally accepted material model and material parameter values for the brain is due to many factors, including study design, participant-specific physiology (Svensson et al. 2021), and the high heterogeneity of brain tissue (Budday et al. 2020). Brain material properties are becoming more widely measured in vivo; in particular, in vivo estimates from multi-frequency MRE including anisotropy are now available (Smith et al. 2022). Different material models may be optimal for different conditions (blast, blunt impact, surgery, brain development) but at corresponding time scales (10s of ms or 10s of Hz) models should be fairly consistent.

The nonlinear transformation of brains from the original anatomical space to the standard MNI-152 space, could, in principle, introduce artificial alterations in the strain fields. However, we found minimal differences between strains before and after registration (see Online Resource Fig. S6). Estimates of strain are affected by inevitable errors from the numerical differentiation of displacement measurements at discrete spatial locations. Noise in the MR signal affects displacement measurements, and the effects of noise are amplified by numerical differentiation.

Elevated strain (occurring at high rate) in brain tissue is widely thought to be the direct cause of injury. Various global strain metrics have been proposed, such as the 95$^{th}$ percentile MPS for time-domain studies involving impact (Zhan et al. 2021; Bayly et al. 2021). The cumulative strain damage measure (CSDM) is another global metric that describes how much of the brain volume has experienced strain surpassing a predetermined threshold of MPS (Knowles and Dennison 2017; Elkin et al. 2019; Wu et al. 2021; Alshareef et al. 2021b; Ji et al. 2022; Barnes-Wood et al. 2024). Such global metrics have three related drawbacks: (**1)** They may represent only the strain in a few elements; (**2)** They do not provide information regarding spatial patterns of strain, orientation of the strain tensor, or the distribution of strain magnitude (Ji et al. 2022), **(3)** They do not account for strain rate effects. The quantitative comparison framework presented here addresses the first two issues. In particular, all 3D strain tensor components at each voxel of the comparison domain are included in the comparisons.

$SM$ is closely related to octahedral shear strain ($OSS$) which is widely used in MRE to assess the magnitude of deformation (see McGarry et al. (2011)). $OSS$ is an estimate of the time-averaged norm of the deviatoric strain tensor. Since brain tissue is nearly incompressible, $OSS$ and $SM$ in the brain are very similar in magnitude and highly correlated. $SM$ is simpler to calculate than $OSS$ for a 3D strain tensor field, as it avoids the need for time averaging and numerical integration by exploiting the voxelwise complex tensor inner product.

In addition to comparisons between simulations and MRE experiments, this framework can be used to compare responses to impulsive loading. Dominant modes of oscillation can be extracted from the time-domain impulse response of the brain (Laksari et al. 2018; Escarcega et al. 2021; Rezayaraghi et al. 2023). Models of the brain's response to impulsive loading can thus, in principle, be evaluated by comparing dominant modes at distinct frequencies to those from experiments.

## 5 Conclusion

This study introduces a framework for comparing model predictions of 3D strain fields during harmonic motion of the head to corresponding experimentally measured strain fields with the same loading. Agreement is described in terms of global strain field correlation values and maps of local correlations between the strain fields. We demonstrated this framework by comparing strain predictions from two models to strain fields in the brains of six human participants. The methods introduced here can be applied to evaluate and improve models that aim to predict 3D strain fields in the intact, living human brain.

## Supplementary Files

This is a list of supplementary files associated with this preprint. Click to download.

- OnlineResource.docx
